# The effect of low glycemic index diet on the reproductive and clinical profile in women with polycystic ovarian syndrome: A systematic review and meta-analysis

**DOI:** 10.1016/j.heliyon.2021.e08338

**Published:** 2021-11-09

**Authors:** Najmieh Saadati, Fatemeh Haidari, Mojgan Barati, Roshan Nikbakht, Golshan Mirmomeni, Fakher Rahim

**Affiliations:** a Fertility, Infertility, and Perinatology, Research Center, Ahvaz Jundishapur University of Medical Sciences, Ahvaz, Iran; b Department of Nutrition Sciences, Nutrition and Metabolic Diseases Research Center, School of Paramedical Sciences, Ahvaz Jundishapur University of Medical Sciences, 61357-15794, Ahvaz, Iran; c School of Medicine, Fertility Infertility and Perinatology Research Center, Ahvaz Jundishapur University of Medical Sciences, Ahvaz, Iran; d Hearing Research Center, Department of Audiology, Ahvaz Jundishapur University of Medical Sciences, Ahvaz, Iran; e Health Research Institute, Thalassemia and Hemoglobinopathies Research Centre, Ahvaz Jundishapur University of Medical Sciences, Ahvaz, Iran

**Keywords:** Glycemic index, Low-GI diets, Endocrine parameters, Blood lipids, PCOSQ domains

## Abstract

**Background:**

Treatment for polycystic ovary syndrome (PCOS) usually initiates with a series of lifestyle modifications such as diet, weight loss, and exercise.

**Aims:**

We, therefore, conducted this meta-analysis to systematically review and evaluate the possible benefits of LGD on a range of anthropometric, clinical, and biochemical parameters in women with PCOS.

**Methods:**

We performed a systematic search through major indexing databases, including Scopus, Pubmed/Medline, ISI web of science, Embase, Cochrane central, and CINAHL (1966–April 30, 2021) using key concepts of PCOS.

**Results:**

Of 935 initial publications, 542 remain after duplicates removal. Then, 141 records were removed at the title and abstract screening level. After excluding 392 literatures, we finally included 8 articles. The final selected studies included 412 overweight and obese individuals with PCOS (207 cases in LGID group and 205 patients in comparators) with a mean age of 21–32 years. Measured emotional health (3 studies, 132 participants, SMD: -1.97; 95%CI:-3.54, -0.40, *P* = 0.01, *I*^*2*^ = 89%) and body hair (3 studies, 132 participants, SMD: -0.40; 95%CI:-0.46, -0.35, *P* < 0.0001, *I*^*2*^ = 0%), were found to be significantly lower in women in LGD vs control diet groups. Moreover, infertility (3 studies, 132 participants, SMD: 1.45; 95%CI: 0.30, 2.61, *P* = 0.01, *I*^*2*^ = 79%) was significantly higher in women in LGD vs control diet groups.

**Conclusion:**

The present meta-analysis has shown that LGD may play a significant role in reducing the risk and improving the clinical and biochemical features of PCOS. So far the evidences for choosing the best dietary modalities for PCOS are not strong to make a definite recommendation.

## 1 Introduction

Polycystic ovary syndrome (PCOS) is a widespread disorder that affects women during the reproductive age and is one of the most common causes of menstrual irregularity, hyper-androgenism, and infertility among young women [1]. PCOS is one of the most common endocrine disorders with cardio-metabolic risk affecting women [2]. This disease is of great importance both clinically and in terms of community health, because its prevalence is very high affecting 18–22% of women at reproductive age [3]. PCOS is a disease of unknown etiology is associated with many clinical symptoms, among which missed or irregular menstruation, ovarian cyst, excessive facial and body hair (hirsutism), and hyperpigmentation [4]. In addition, the disease includes a set of other symptoms such as excessive confusion and depression [5].

Endocrine disruption and increasing androgens such as testosterone and dehydroepiandrosterone (DHEA) associated with PCOS could have a negative impact on ovarian function and follicular development and growth; therefore, PCOS can lead to infertility, or increasing endometrial hyperplasia and cancers [6]. Another factor is the increased chance of developing diseases, especially in older age, such as obesity, insulin resistance, type 2 diabetes, high blood pressure, and impaired lipid status or uterine cancer [7, 8]. In PCOS increasing the pulsatility of luteinizing hormone (LH) levels regarding both frequency and amplitude, increases theca cell production of androgens, while relatively low FSH secretion impairs follicle maturation and consequently ovulation [9]. Thus, the increasing incidence of PCOS among young women of reproductive age and its complications, such as infertility, has necessitated further studies on this disease.

Treatment for PCOS usually initiates with a series of lifestyle modifications such as diet, weight loss, and exercise. Losing weight is one of the most effective measures to regulate the menstrual cycle and improve the symptoms of PCOS [10]. In low glycemic diets (LGD), the glycemic index (GI) is used to determine which foods have the least significant effect on blood sugar levels; thus, LGD may help weight loss [11].

Previous evidence found that a LGD may have benefits for individuals undergoing in vitro fertilization (IVF) [12] and natural fertility [13]. Other studies revealed that LGD reduced body fat and BMI and improved pregnancy outcomes [14, 15]. So far several meta-analyses have assessed the effect of nutritional intervention on many aspects of PCOS such as insulin resistance [16, 17, 18, 19, 20, 21], Biochemical parameters [22, 23, 24], and androgenic profiles [25, 26]. We, therefore, conducted this meta-analysis to systematically review and evaluate the possible benefits of LGD on a range of anthropometric, clinical, and biochemical parameters in women with PCOS.

## 2 Methods

This meta-analysis was conducted following Preferred Reporting Items for Systematic Reviews and Meta-analyses (PRISMA) guidelines [27].

### 2.1 Search strategy

We comprehensively searched major indexing databases, including Scopus, Pubmed/Medline, ISI web of science, Embase, Cochrane central, and CINAHL using keywords: (“polycystic ovary syndrome” OR “PCOS”) AND ("Glycemic Index"[Mesh] OR "Glycemic Load"[Mesh] OR "Dietary Carbohydrates"[Mesh] OR "Dietary Sucrose"[Mesh] OR "Dietary Sugars"[Mesh] OR “GI” OR “GL” OR "Diet, Diabetic"[Mesh]) from Jan 1, 1980, to April 26, 2021, with no language restrictions. Also, two major clinical trial registries, including clinicaltrials.gov and the WHO clinical trials search portal were searched. We also performed hand searching through collections of records cited by the formerly found articles.

### 2.2 Inclusion and exclusion criteria

All randomized clinical trials (RCTs) of I week or longer in people with PCOS, using LGD compared with the control diet, measuring anthropometric, clinical, and biochemical parameters were included. Review articles, animal studies, letter to the editors, commentaries, case reports, observational studies, using LGD in non-PCOS people, were excluded.

### 2.3 Outcome measures

We collected information regarding all outcome measures. The first outcome of interest includes the assessment of anthropometric measures. Second outcomes were cardio-metabolic risk profile and biochemical assessments, including Serum total cholesterol (TC) and triglyceride (TG), Serum high-density lipoprotein cholesterol (HDL-C), Serum low-density lipoprotein cholesterol (LDL-C) levels, TC/HDL ratio, luteinizing hormone (LH), follicle-stimulating hormone (FSH), total testosterone (TT), sex-hormone binding globulin (SHBG), dehydroepiandrosterone sulfate (DHEA-S), and prolactin. Third and most important outcomes were quality of life (QOL) according to the PCOS Health-Related Quality of Life Questionnaire (PCOSQ) in term of five domains, including emotional health, body hair, infertility, weight, and menstrual problems scoring range from 1 (a lower QOL) to 7 (a higher QOL).

### 2.4 Study selection

Two authors (FR and FH) independently performed the title and abstract screening. Any disagreement was resolved either by double-check the reference paper or discussion with a third author.

### 2.5 Methodological quality assessment

Two authors (FR and FH) independently conducted the methodological quality assessment with special consideration to potential sources of risk of bias. We used the Cochrane Collaboration's quality assessment tool for risk of bias assessment in RCTs [28]. Any disagreement was resolved either by double-check the reference paper or discussion with a third author. We rated the quality of the evidence as low to moderate according to GRADE criteria, as most information is from studies judged to be at unclear risk of bias [29].

### 2.6 Data extraction

Data extraction was performed by one reviewer (FH) and double-checked by another author (NS). Authors extracted data, including author's name, publication year, country, intervention, comparators, and outcomes of interest. In case the outcomes of interest were missing, we contact the authors three times; Also, if the outcomes were only presented in figures, we used WebPlotDigitizer to extract the data [30]. Median and range were converted to mean and standard deviation (SD) using the standard formula.

### 2.7 Data analysis

We used RevMan 5.3 software for data analysis, as well as used standardized mean difference as effect size. If data were present as median and range, we used Wan *et al.* methods to estimate the mean and standard deviation [31]. The Biochemical units such as LDL and TC were transformed from mg/dL to mmol/L as appropriate. Heterogeneity was described as the total variability (*I*^*2*^). The significant heterogeneity was tested by *χ*^2^ test. Low heterogeneity was indicated as *I*^*2*^ < 40%. In case the heterogeneity was significant (*I*^*2*^ > 75%), the source of heterogeneity was detected before meta-analysis. We conducted sub-group analyses based on various comparators. To assess publication bias we used funnel plots.

## 3 Results

### 3.1 Characteristics of included studies

**Figure 1:**
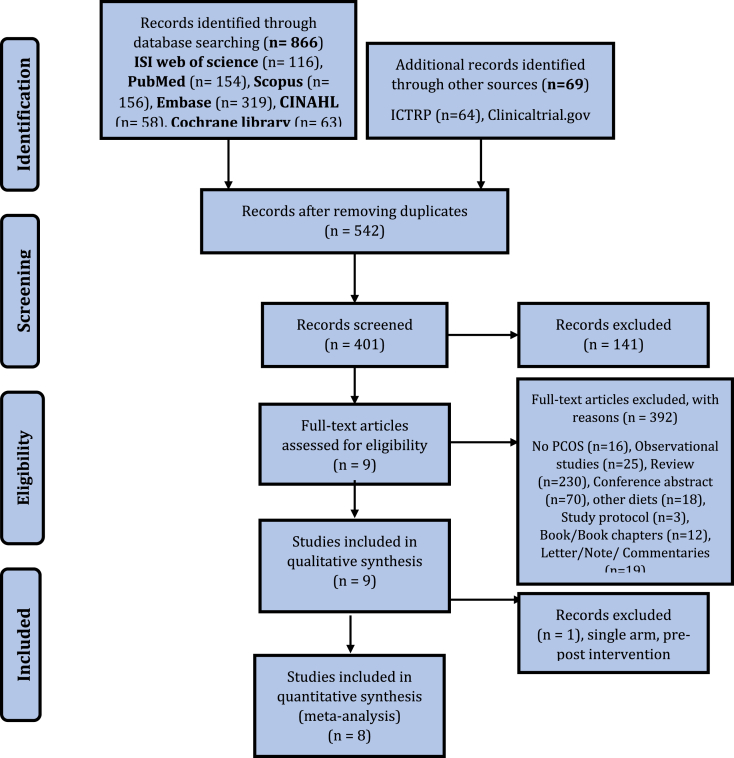
PRISMA flow diagram.

Of 935 initial publications, 542 remain after duplicates removal. Then, 141 records were removed at the title and abstract screening level. After excluding 392 literatures, we finally included 9 articles. We then removed Bar *et al.* study due to missing comparators [32]. Finally, eight articles were included in qualitative and quantitative analyses (Figure 1). The final selected studies included 412 overweight and obese individuals with PCOS (207 cases in the LGD group and 205 patients in comparators) with a mean age of 21–32 years (Table 1) [33-40]. Studies varied in length from 3 to 12 months, comparing LGID against high glycemic index diet, low-calorie diet, normal glycemic diet, low-fat diet, therapeutic lifestyle changes, and conventional hypocaloric diet.

**Table 1:** Characteristics of included studies.

Study ID	Country	Study type length	No. of patients		BMI category	Age (Year)	PCOS diagnosis	Intervention type	Comparators	Outcome	Quality of evidence
			G1	G2							
Marsh *et al.* 2010 [35]	Australia	Parallel 3 months	50	46	Overweight and obese	18–40	Rotterdam criteria [41]	LGID	CHD	The beneficial rule of LGID in the management of PCOS	Low∧^%^
Mehrabani *et al.* 2012 [36]	Iran	Parallel 3 months	30	30	Overweight and obese	20–40	Rotterdam criteria and mF-G score [42]	LGID + MHCD	CHCD	Significantly led to reduced body weight and androgen levels	Moderate^%^
Asemi *et al.* 2014 [33]	Iran	Parallel 3 months	27	27	Overweight and obese	18–40	Rotterdam criteria and mF-G score	LGID DASH-style diet	CD	Significantly reduction in LDL and increase in TAC and GSH levels	Moderate^%^
Panico *et al.* 2014 [37]	Italy	Cross-over 3 months	15	15	Overweight	18–40	Rotterdam criteria	LGID	HGID	Improves insulin resistance and serum androgen levels	High
Turner-McGrievy *et al.* 2014 [39]	USA	Parallel 6 months	9	9	Overweight and obese	18–35	Rotterdam criteria	LGID-vegan	LCD	Effective for promoting short-term weight loss	Moderate^%^
Sordia-Hernández *et al.* 2016 [38]	Mexico	Parallel 3 months	20	20	Overweight	18–35	Rotterdam criteria	LGID	NGID	Improves insulin resistance and serum androgen levels	Low∧^%^
Wong et al. 2016 [40]	USA	Parallel 3 months	9	10	Overweight and obese	13–21	Rotterdam criteria	LGID	LFD	Beneficial for weight control but did not attenuate biochemical hyperandrogenism	Moderate^%^
Kazemi et al. 2019 [34]	Canada	Parallel 12 months	47	48	Overweight and obese	18–35	Rotterdam criteria	LGID pulse-based diet,	TLC	Improve cardio-metabolic disease risk factors	High

LGID, Low glycemic index diet; CHD, conventional healthy diet; CHCD, conventional hypocaloric diet; MHCD, modified hypocaloric diet; mF-G score, Modified Ferriman–Gallwey score; DASH, Dietary Approaches to Stop Hypertension; CD, Control diet; insulin, triglycerides and low-density lipoprotein cholesterol (LDL-c); TAC, plasma total antioxidant capacity; GSH, total glutathione; HGID, High glycemic index diet; LCD, Low calorie diet; NGID, normal glycemic diet; LFD, Low fat diet; TLC, Therapeutic Lifestyle Changes; Factors downgrading any specific evidence: ∗ Limitations (risk of bias), $ Inconsistency of results, # indirectness of results, % Imprecision, ^ Publications bias; **GRADE of evidence: High quality:** Further research is very unlikely to change our confidence in the estimate of effect. **Moderate quality:** Further research is likely to have an important impact on our confidence in the estimate of effect and may change the estimate. **Low quality:** Further research is very likely to have an important impact on our confidence in the estimate of effect and is likely to change the estimate. **Very low quality:** We are very uncertain about the estimate.

### 3.2 Quality of included studies

**Figure 2:**
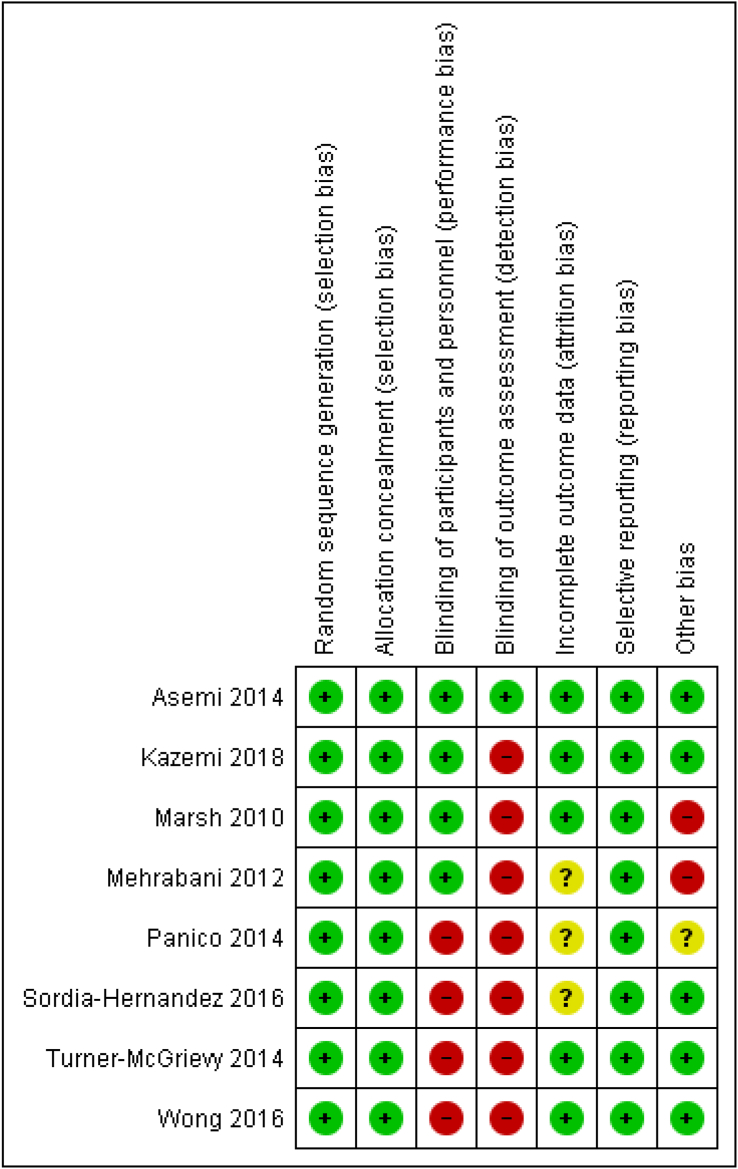
The risk of bias of included studies.

We found a substantial risk of bias mostly due to the wide-ranging problems of blinding. The method of randomization was mostly described (Figure 2).

**Glycemic and insulinemic control:** We observed a statistically significant lowering of 2-hour insulin (4 studies, 177 participants, SMD: -0.79; 95%CI:-1.33, -0.24, *P* = 0.005, *I*^*2*^ = 57%) and HOMA-IR (6 studies, 293 participants, SMD: -0.36; 95%CI:-1.33, -0.24, *P* = 0.005, *I*^*2*^ = 57%) as a consequence of LGD compared to control diets (Table 2). We also did not find any statistically significant differences in terms of fasting glucose (FG), fasting insulin (FI), 2-hour glucose, HbA1c, and HOMA2-IS between LGD and control diets (Table 2).

**Table 2:** Outcomes of meta-analyses (SMD [95% CI).

Outcome	Studies	Participants	Model	I^2^	p-values	Effect Estimate
Insulin sensitivity measures, endocrine parameters, and lipid profile						
Fasting Glucose (mmol/L)	7	280	Random	96%	0.27	-0.79 [-2.17, 0.60]
2-hour glucose (mmol/L)	4	177	Random	92%	0.16	-0.92 [-2.22, 0.38]
Fasting Insulin (μIU/mL)	5	192	Random	52%	0.47	-0.16 [-0.60, 0.28]
**2-hour insulin (μIU/mL)**	**4**	**177**	**Random**	**57%**	**0.005**	**-0.79 [-1.33, -0.24]**
**HOMA2-IR**	**6**	**293**	**Fixed**	**0%**	**0.003**	**-0.36 [-0.59, -0.12]**
HOMA2-IS (%)	2	138	Fixed	0%	0.13	-0.26 [-0.59, 0.08]
HbA1c (%)	2	114	Random	80%	0.65	0.27 [-0.90, 1.44]
**LDL-c (mmol/L)**	**5**	**307**	**Fixed**	**0%**	**0.05**	**-0.22 [-0.01, -0.45]**
**TG (mmol/L)**	**6**	**337**	**Fixed**	**0%**	**0.009**	**-0.02 [-0.50, -0.07]**
**Total cholesterol (TC) (mmol/L)**	**6**	**337**	**Random**	**68%**	**0.04**	**-0.48 [-0.11, -1.07]**
**HDL-c (mmol/L)**	**5**	**307**	**Random**	**59%**	**<0.0001**	**-0.92 [-0.53, -1.31]**
**TC/HDL-c**	**4**	**177**	**Fixed**	**0%**	**0.03**	**-0.07 [-0.14, -0.01]**
hsCRP (mg/L)	4	178	Fixed	9%	0.54	0.09 [-0.20, 0.39]
Fasting insulin/glucose ratio	4	190	Random	88%	0.46	-0.37 [-1.34, 0.61]
Total insulin AUC (μIU/ml∗ min)	2	80	Random	81%	0.92	0.07 [-1.16, 1.29]
Total glucose AUC (mmol/L∗ min)	2	80	Random	82%	0.83	0.14 [-1.15, 1.42]
Incremental glucose AUC (mmol/L∗ min)	2	80	Random	70%	0.57	0.28 [-0.69, 1.25]
**SHBG (nmol/L)**	**3**	**117**	**Random**	**67%**	**0.04**	**-0.72 [-1.42, -0.02]**
**FAI (%)**	**2**	**98**	**Random**	**0%**	**<0.0001**	**1.16 [0.72, 1.59]**
**LH (IU/L)**	**4**	**215**	**Fixed**	**27%**	**0.03**	**-0.30 [-0.57, -0.03]**
**FSH (IU/L)**	**4**	**215**	**Fixed**	**0%**	**0.0002**	**0.19 [-0.65, 1.04]**
**Testosterone (ng/dl)**	**6**	**329**	**Random**	**42%**	**0.0009**	**-0.52 [-0.83, -0.22]**
Androstenedione (A4) (ng/dl)	2	63	Fixed	0%	0.73	0.09 [-0.41, 0.58]
DHEAS (ng/ml) or ug/dl	4	193	Random	78%	0.34	0.33 [-0.35, 1.00]
Prolactin (ng/ml)	3	165	Random	68%	0.16	-0.43 [-1.02, 0.17]
Anthropometrics, body composition measures, and physiologic measures						
BMI (kg/m2)	8	258	Fixed	22%	0.83	-0.03 [-0.27, 0.22]
Weight (kg)	8	298	Random	83%	0.17	-0.43 [-1.05, 0.18]
**Waist circumference (cm)**	**2**	**110**	**Fixed**	**0%**	**0.002**	**-6.16 [-10.12, -2.20]**
Trunk fat mass (kg)	2	80	Random	76%	0.77	0.16 [-0.93, 1.25]
Percent body fat (%)	3	129	Random	78%	0.77	0.52 [-2.99, 4.03]
**Total body fat mass (kg)**	**2**	**110**	**Fixed**	**0%**	**<0.0001**	**-2.90 [-3.63, -2.17]**
Systolic blood pressure (mm Hg)	2	80	Fixed	0%	1.00	0.00 [-0.44, 0.44]
Diastolic blood pressure (mm Hg)	2	80	Random	46%	0.72	-0.77 [-4.99, 3.44]
Total body lean mass (kg)	2	110	Fixed	0%	0.06	-0.37 [-0.75, 0.01]
Dietary parameters						
Energy (kcal/day)	7	258	Random	82%	0.19	-0.44 [-1.11, 0.22]
Fat (% of total Energy)	6	240	Random	53%	0.40	0.17 [-0.23, 0.56]
Total fat (g)	3	123	Random	78%	0.50	0.30 [-0.58, 1.18]
Protein (% of total energy)	6	240	Random	94%	0.10	1.08 [-0.20, 2.36]
Total protein (g)	3	123	Random	87%	0.08	-1.14 [-2.44, 0.15]
**Carbohydrate (% of total energy)**	**7**	**258**	**Random**	**92%**	**0.009**	**-1.44 [-2.52, -0.35]**
Total carbohydrate (g)	3	123	Fixed	39%	0.78	0.05 [-0.31, 0.41]
**Glycemic index**	**4**	**143**	**Random**	**95%**	**0.04**	**-2.87 [-5.58, -0.16]**
**Glycemic load**	**4**	**143**	**Random**	**91%**	**0.04**	**-1.58 [-3.09, -0.06]**
Saturated fatty acid (SFA) gr/day	4	141	Random	96%	0.26	-1.66 [-4.53, 1.22]
MUFA (g/day)	3	124	Fixed	32%	0.11	0.27 [-0.22, 0.79]
PUFA (g/day)	4	163	Random	96%	0.10	1.68 [-0.30, 3.65]
SFA (g/day)	4	197	Random	96%	0.003	-0.49 [-0.81, -0.17]
Fiber (g)	5	191	Random	90%	0.02	2.81 [0.42, 5.21]
**Cholesterol intake (mg/day)**	**6**	**231**	**Random**	**84%**	**0.01**	**-0.76 [-1.48, -0.046]**
Clinical outcomes (Change in PCOSQ domains)						
**Emotional health**	**3**	**132**	**Random**	**89%**	**0.01**	**-1.97 [-3.54, -0.40]**
**Body hair**	**3**	**132**	**Fixed**	**0%**	**<0.0001**	**-0.40 [-0.46, -0.35]**
**Fertility**	**3**	**132**	**Random**	**79%**	**0.01**	**1.45 [0.30, 2.61]**
Menstrual concerns	3	132	Random	96%	0.19	-1.58 [-3.94, 0.77]

**Endocrine parameters:** Total testosterone level improved significantly (reduced) in LGD group against comparators with a SMD of -0.52 (95% CI: -0.83 to -0.22, *P* = 0.0009, *I*^*2*^ = 42%). Also, LH levels were improved significantly (reduced) in the LGD group compared with comparator groups, with a SMD of -0.30 (95% CI: -0.57 to -0.03, *P* = 0.03, *I*^*2*^ = 27%). Measures of FSH levels were found to be improved significantly (decreased) in LGD group vs. comparator groups, with an SMD of -0.52 (95% CI: -0.79 to -0.25, *P* = 0.0002, *I*^*2*^ = 0%). SHBG levels were increased non-significantly in women with PCOS who used LGD intervention vs. comparator groups, with a SMD of 0.6 (95% CI: -0.14 to 1.34, *P* = 0.11, *I*^*2*^ = 77%). Measures of DHEA-S levels were found to be improved non-significantly (increased) in LGD group vs. comparator groups, with an SMD of 0.33 (95% CI: -0.35 to 1.00, *P* = 0.34, *I*^*2*^ = 78%). Prolactin levels were decreased non-significantly in women with PCOS used LGD intervention vs. comparator groups, with a SMD of -0.43 (95% CI: -01.02 to 0.17, *P* = 0.16, *I*^*2*^ = 68%) (Table2).

**Lipid profile:** We observed a statistically significant lowering TC (6 studies, 337 participants, SMD: -0.48; 95%CI:-0.11, -1.07, *P* = 0.04, *I*^*2*^ = 68%), TG (6 studies, 337 participants, SMD: -0.02; 95%CI:-0.50, -0.07, *P* = 0.04, *I*^*2*^ = 0%), LDL (5 studies, 307 participants, SMD: -0.22; 95%CI:-0.01, -0.45, *P* = 0.05, *I*^*2*^ = 0%), and HDL (5 studies, 307 participants, SMD: -0.92; 95%CI:-0.53, -1.31, *P* < 0.0001, *I*^*2*^ = 59%) as a consequence of LGD compared to control diets (Table 2).

**Anthropometrics and body composition measures:** Overall, the included studies failed to confirm a statistically significant difference between LGD and control diets in lowering anthropometrics and body composition measures (Table 2). Only measures that decreased significantly were waist circumference (2 studies, 110 participants, SMD: -6.16; 95%CI:-10.12, -2.20, *P* = 0.002, *I*^*2*^ = 0%) and total body fat mass (2 studies, 110 participants, SMD: -2.90; 95%CI:-3.63, -2.17, *P* < 0.0001, *I*^*2*^ = 59%) (Table 2).

**Physiologic measures:** Overall, the included studies failed to confirm a statistically significant difference between LGD and control diets in lowering systolic and diastolic blood pressure (Table 2).

Dietary parameters: We found a superior lowering carbohydrate (7 studies, 258 participants, SMD: -1.44; 95%CI:-2.52, -0.35, *P* = 0.009, *I*^*2*^ = 92%), GI (4 studies, 143 participants, SMD: -2.87; 95%CI:-5.58, -0.16, *P* = 0.04, *I*^*2*^ = 95%), GL (4 studies, 143 participants, SMD: -1.58; 95%CI:-3.09, -0.06, *P* = 0.04, *I*^*2*^ = 91%), and cholesterol intake (6 studies, 231 participants, SMD: -0.76; 95%CI:-1.48, -0.046, *P* = 0.01, *I*^*2*^ = 84%) as a consequence of LGD compared to control diets (Table 2).

**Clinical outcomes (Change in PCOSQ domains)**: Measured emotional health (3 studies, 132 participants, SMD: -1.97; 95%CI:-3.54, -0.40, *P* = 0.01, *I*^*2*^ = 89%) and body hair (3 studies, 132 participants, SMD: -0.40; 95%CI:-0.46, -0.35, *P* < 0.0001, *I*^*2*^ = 0%), were found to significantly lower in women in LGD vs control diet groups. Moreover, fertility (3 studies, 132 participants, SMD: 1.45; 95%CI: 0.30, 2.61, *P* = 0.01, *I*^*2*^ = 79%) was significantly higher in women in LGD vs control diet groups (Table 2). Though menstrual concerns were lower in women who received LGD vs control diet, the estimation was not statistically significant.

### 3.3 Sensitivity analyses

**Figure 3:**
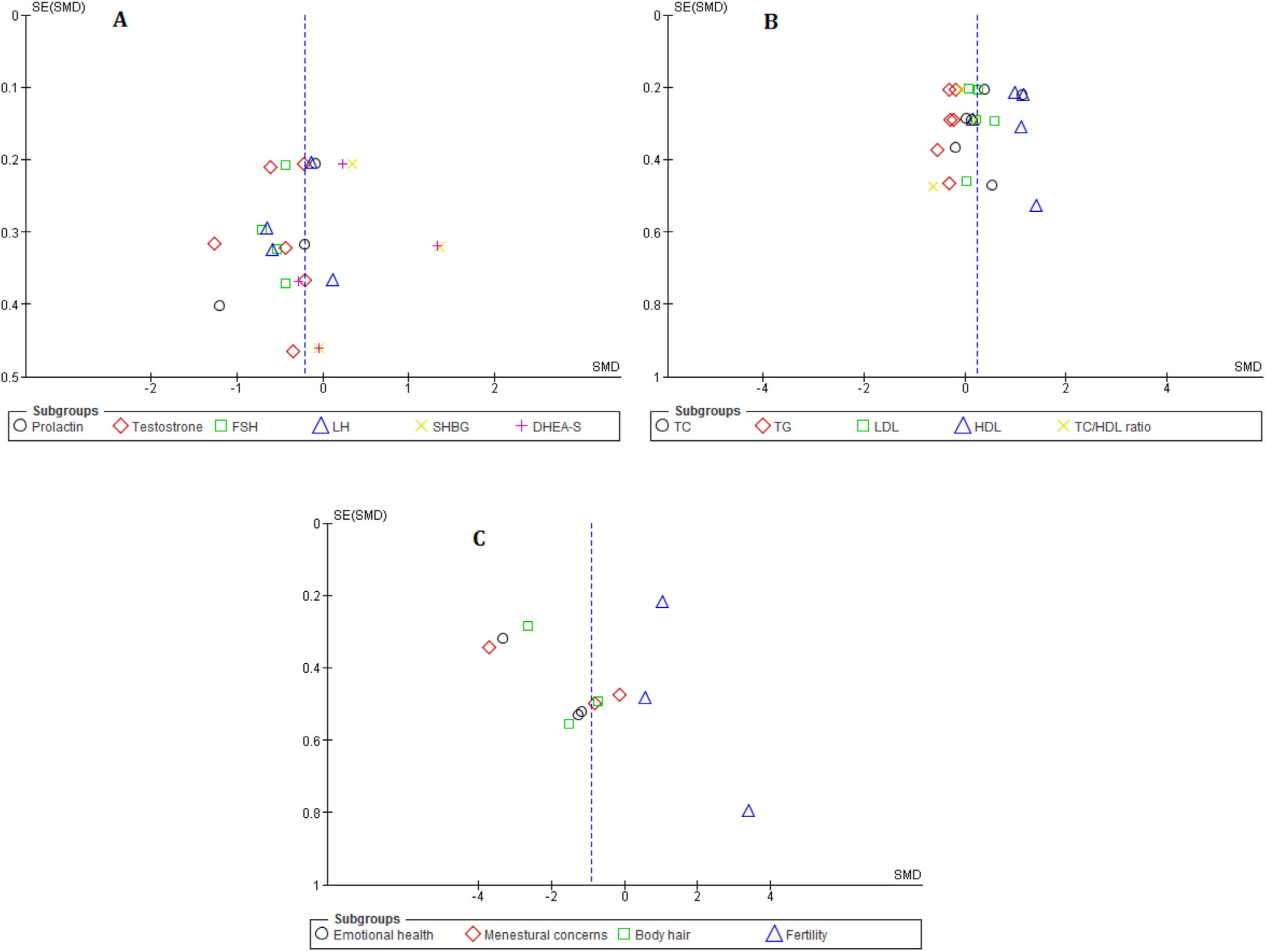
Publication bias funnel plots for the effect of low glycemic diet on endocrine parameters, and lipid profile (A and B) and clinical outcomes (Change in PCOSQ domains) in patient with PCOS.

There was a substantial variation in the comparators of included studies, thus, we performed a sensitivity analysis of the effects of various comparator diets on study outcomes. We found no statistically significant differences between LGD and control diets in the sensitivity analysis. We did not observe any publication bias using funnel plots for insulin sensitivity measures, endocrine parameters, lipid profile, and clinical outcomes (Change in PCOSQ domains), **in which a**ll plots appeared to be symmetrical, with no obvious publication bias (Figure 3).

## 4 Discussion

The present meta-analysis has shown that LGD can play a significant role in reducing the risk and improving the clinical and biochemical features of PCOS. The best treatment for PCOS is yet to be recognized, but evidence supports a multifactorial approach, including any combination of one or more of the interventions such as diet and lifestyle management, use of pharmaceuticals (oral contraceptives and cyclic progestins, antiestrogens, gonadotropins, and insulin sensitizers), and surgery. In one evaluation of 138 endocrinologists and 172 gynecologists that was carried out by Cussons *et al*, the majority of respondents recommended that the first line of treatment for all presentations of PCOS should be diet and exercise [43].

As a whole, the two primary strategies for controlling the disease include weight control, insulin production, and insulin resistance [44]. Generally, insulin resistance is present in both obese and non-obese patients with PCOS [45]. Because insulin plays an important role in PCOS and its metabolic features, controlling the disease with diet is the best step for people who want to manage PCOS and its associated symptoms [46, 47]. Approximately, 50% of people with PCOS develop diabetes or pre-diabetes before turning 40 years age [48]; therefore, following a special lifestyle and diets that maintain body weight and eventually increases good insulin levels, reduces the risk of the disease and its associated adverse events [49, 50, 51, 52, 53, 54].

Hence, the recommended diet composition for PCOS patients is drawn from the available recommendations for the dietary management of type 2 diabetes. Of note, carbohydrates have been reported to influence PCOS primarily via their impact on insulin concentrations in the blood. Studies demonstrate that a high-fiber, low-glycemic-index diet such as the DASH diet will result in an overall weight loss and a reduction in insulin resistance [33, 55]. A 6-month, low-carbohydrate, ketogenic diet was used by Mavropoulos *et al* to investigate the influence of a low intake of carbohydrates on obese and overweight patients with PCOS. They observed the amelioration in weight, free testosterone, LH: FSH ratio, and fasting insulin [56]. Serum androgens were also reported to be lowered in individuals who had been consuming a high-fat diet, then began taking an isocaloric, high-fiber, low-fat diet for 8 weeks [57].

Proper diets for weight loss and improving the concentration of lipid and metabolic profiles place great emphasis on the effects of dietary carbohydrates and fats. The results of studies indicate that if the fat intake is reduced from the total energy intake, the consumption of carbohydrates will increase [58, 59]. Patients with PCOS, just like other people must eat, therefore, following special diets such as LGD with potentially positive effects on blood glucose and insulin may reduce the risk of the disease and its associated adverse events [60]. In this context, the improvements were seen in insulin and HOMA-IR may suggest a potential improvement in insulin sensitivity following the use of LGD. In line with our findings, a meta-analysis was conducted by Zafar *et al.* to evaluate the hypothesis that LGD may lead to lowering measures of blood glucose control in individuals with both types of diabetes. They suggested a potential improvement in insulin sensitivity with LGD compared with other dietary interventions [61].

Recently, Kazemi *et al* conducted a similar systematic review and meta-analysis of RCTs to review evidence on the effects of GI or GL index diet on cardio-metabolic and reproductive profiles of women with PCOS [62]. They included seven RCTs, including cardio-metabolic and reproductive profiles. Our meta-analysis included eight RCTs and presents clinical outcomes based on PCOSQ domains, including emotional health, body hair, menstrual concerns, and fertility. Having PCOS does not mean the patient can't get pregnant, because this syndrome is among the most common, but treatable, causes of infertility in women. In women with PCOS, the hormonal imbalance interferes with ovulation, so PCOS significantly increases the risk of infertility [63]. The earlier interventions have failed or can be offered as a treatment of the first choice in selective causes that impair the occurrence of pregnancy for the woman with PCOS; therefore, choosing an effective treatment option addresses not only subfertility, anovulation, and fertility plays an important role as a holistic approach [64].

Overall, whether in combination with pharmacotherapy or as a standalone treatment, diet and lifestyle modifications should be recommended in the treatment of women with PCOS. Women with PCOS who are overweight should lose weight through diet and exercise. Their diet should contain high-fiber complex carbohydrates, moderate levels of protein, and a sufficient amount of fat to meet essential fatty acid requirements. It should also focus on omega-3 fatty acids and monounsaturated fatty acids and contain limited amounts of trans and saturated fats, as recommended by available evidence. These dietary modifications should significantly ameliorate many of the symptoms that co-exist with PCOS. Because symptoms of PCOS usually occur with menarche, it is also essential that adolescents who are at risk be screened, and dietary modifications for the prevention of PCOS and related comorbidities be carried out.

### 4.1 Limitations

This study contains some limitations. There was insufficient data available for any outcome to see if there was a dose-response relationship e.g. the greater the difference in GI between the low GI and the control, the greater the effect on the outcome. Also, there was insufficient evidence to determine how soon the effects were seen, or if longer interventions were more effective.

## 5 Conclusion

The present meta-analysis has shown that LGD may play a significant role in reducing the risk and improving the clinical and biochemical features of PCOS. PCOS has a negative impact on the general health of many women, although most women who suffer from this metabolic disease came to the doctors for treatment of menstrual irregularity, hirsutism, and infertility. As a health-care provider, the ultimate goal for the treatment of PCOS should be the improvement of fundamental health issues and consequently general health status. Also, other goals include preventing long-life complications of metabolic disorders due to PCOS like increased cardio-metabolic risks or obesity, instead of just treatment of disease symptoms. In this manner, if we could find interventions that improve general health status, as the consequence, symptoms like menstrual irregularity, hirsutism and infertility might get better or even be cured. With such dietary modalities, the risk of endometrial cancer might be omitted. So far the pieces of evidence for choosing the best diet modalities for PCOS are not strong to make definite recommendations.

## Declarations

### Author contribution statement

Fakher Rahim: Conceived and designed the analysis; Performed the experiments; Contributed reagents, materials, analysis tools or data; Wrote the paper.

Fatemeh Haidari and Najmieh Saadati: Conceived and designed the experiments; Performed the experiments.

Mojgan Barati and Roshan Nikbakht: Contributed reagents, materials, analysis tools or data; Wrote the paper.

Golshan Mirmomeni: Analyzed and interpreted the data.

### Funding statement

This work was supported by "deputy of research and technology affairs of Ahvaz Jundishapur University of Medical Sciences (Reference number: FIRC-0017, Ethics approval Code: IR.AJUMS.REC.1400.329)".

### Data availability statement

Data included in article/supplementary material/referenced in article.

### Declaration of interests statement

The authors declare no conflict of interest.

### Additional information

No additional information is available for this paper.
